# The experiences of living with dementia within an under-served geographical area: a systematic review and qualitative synthesis

**DOI:** 10.1093/ageing/afag190

**Published:** 2026-06-24

**Authors:** Jessica Kay, Greta Rait, Alice Burnand, Abi Woodward, Thomas Littlejohns, Megan Armstrong

**Affiliations:** Research Department of Primary Care and Population Health, UCL Medical School (Royal Free Campus), University College London, London, United Kingdom of Great Britain and Northern Ireland; Research Department of Primary Care and Population Health, UCL Medical School (Royal Free Campus), University College London, London, United Kingdom of Great Britain and Northern Ireland; Research Department of Primary Care and Population Health, UCL Medical School (Royal Free Campus), University College London, London, United Kingdom of Great Britain and Northern Ireland; Centre for Psychiatry and Mental Health, Wolfson Institute of Population Health, Queen Mary University of London, London, United Kingdom of Great Britain and Northern Ireland; Nuffield of Department of Population Health, University of Oxford, Oxford, United Kingdom of Great Britain and Northern Ireland; Research Department of Primary Care and Population Health, UCL Medical School (Royal Free Campus), University College London, London, United Kingdom of Great Britain and Northern Ireland

**Keywords:** qualitative research, systematic review, underserved areas, dementia, older people

## Abstract

**Background:**

Dementia affects millions globally and presents diverse challenges shaped by personal, social and environmental factors. People living in under-served rural, coastal and deprived communities often face additional barriers to diagnosis, care and support, limiting person-centred approaches. These inequalities can negatively impact quality of life, social inclusion and health outcomes. Whilst dementia experiences have been widely studied, the influence of geographical context remains underexplored. Understanding this is essential to improving equitable, person-centred care across diverse settings.

**Methods:**

MEDLINE, PsycInfo, Cochrane Library and Web of Science were searched from inception in February 2025 for qualitative studies on people’s experiences of living with dementia in rural, coastal or deprived areas. The review was not limited by country or date. Data were coded and thematically synthesised using NVivo.

**Findings:**

Seventy-three full texts were screened using Rayyan and 15 studies were included in the review. Thirteen studies were based in rural areas and two in deprived areas. No included studies were set in coastal areas. Four analytical themes were developed: navigating stigma, privacy and disclosure***,*** navigating fragmented healthcare systems and services, lack of appropriate and accessible services and positive experiences of managing dementia.

**Discussion:**

Key barriers to managing dementia included limited service availability, unsuitable support, stigma and logistical challenges. Findings underscored the need for person-centred, context-sensitive care that considers geographic, social and cultural factors. Future research should further explore diverse under-served settings to inform equitable dementia care particularly in deprived and coastal areas.

## Key Points

- People with dementia in rural and deprived areas face persistent challenges to managing dementia.
- There is a desperate need for more place sensitive services that align with local values.
- Key barriers to managing dementia included limited-service availability, unsuitable support, stigma and logistical challenges.

## Introduction

Dementia can significantly impact individuals’ ability to make decisions, their relationships with family members and friends, and their sense of identity [1]. These challenges can be exacerbated within under-served areas, where population needs exceed the availability or accessibility of health and social care services. Factors such as deprivation, rurality, coastal location, workforce shortages and transport or infrastructure barriers contribute to unequal access to diagnosis and post-diagnostic support [2, 3].

The UK Living Well with Dementia strategy positions equity of access and post-diagnostic support as central to improving quality of life for people living with dementia [4]. However, such access is unevenly distributed. In rural areas, healthcare services face a lack of funding, few healthcare practitioners to cover large areas and poor connectivity (e.g. transportation) [5]. For rural-dwelling people with dementia, this can lead to issues with receiving a timely diagnosis or misdiagnosis [6], a higher number of hospital admissions [7] and higher rates of dementia-related mortality [8]. Similarly, living in an area of high deprivation has been associated with decreased cognition and increased risk of dementia [9, 10], later-stage diagnoses [11], increased hospital admissions [7] and reduced life expectancy [12].

Living by the coast is linked to lower healthy life expectancy and disability-free life expectancy, compared to non-coastal areas [13]. Little research explores the experiences of managing dementia within coastal areas. However, as of 2020, 67% of coastal areas experienced high income deprivation [14] and several coastal areas experienced a lack of suitable and affordable housing, causing challenges to managing health [15]. It can be inferred that similar challenges to managing dementia in deprived areas may also be evident in coastal areas, yet how these experiences are shaped by location is not explicitly stated within existing literature.

Several studies have explored the link between location and how people with dementia navigate daily life [16, 17] however, these studies tend to focus on only one area. The lived experiences of people with dementia within and across these settings can vary significantly, shaped by differences in availability of transportation, formal and informal support, engagement with the community and geographical surroundings. Furthermore, intersections such as ethnicity, gender, age and sexual orientation have also been found to influence individuals’ experiences of living with dementia [18].

The aim of this review is to explore and synthesise the experiences of living with dementia in under-served areas, to highlight barriers or facilitators to accessing support and to identify how place shapes lived experiences of dementia. This review will draw on experiences of people living with dementia that are articulated by themselves as well as informal carers and health professionals.

## Methods

This systematic review employs a thematic synthesis methodology. The review is reported according to the latest Preferred Reporting Items for Systematic Reviews and Meta-Analysis (PRISMA) guidelines [19].

The review protocol is registered on the PROSPERO database (24 February 2025, CRD420250645461).

### Inclusion/exclusion criteria

Studies were included if they:

- Focused on any perspectives, experiences, barriers or facilitators of managing dementia for people living with dementia.
- Were conducted in deprived, rural or coastal areas, as defined by the authors of the reviewed studies.
- Included adults (≥18) with a dementia diagnosis (any type), informal (or unpaid) carers, stakeholders or health care professionals who worked with people with dementia.
- Used any qualitative design.

Studies were excluded if they:

- Focused on the risk of developing dementia or mild cognitive impairment.
- Researched only informal carers’ lived experiences of being a carer for someone with dementia, or if this could not be separated from experiences of people with dementia.
- Were dissertations/theses, abstracts, review articles or conference papers.
- Were not available in English.

### Search strategy and data extraction

Database searches were conducted in February 2025 of Web of Science, PsycInfo, MEDLINE (Ovid) and the Cochrane Library from inception, with no restrictions. The search strategy combined dementia-related terms with terms relating to geographical areas (e.g. rural, deprived, coastal). The full MEDLINE strategy is provided in Appendix 1 of the Supplementary Data section. The screening process was conducted using Rayyan. Titles and abstracts were screened by the lead reviewer (JK), with 50% dual screened by a second reviewer (AB). All full-text papers were dual screened by JK and either AB or MA and disagreements were resolved through full team discussion. Grey literature sources were checked to see if they had generated any peer-reviewed journal articles. Reference lists of included studies were also screened for additional relevant papers.

A data-extraction table was developed and piloted by JK and MA. JK extracted data on author details, aims, area detail, research design, participants and key findings which was then checked for accuracy by MA.

### Quality assessment

This review used the qualitative Joanna Briggs Institute (JBI) quality checklist to appraise all included studies [20]. The checklist consists of ten questions which assess the congruity of the methodology, the influence of the researcher(s), ethical considerations and the flow of the research [20]. JK and AB coded each criterion independently as ‘yes’ = 1 and ‘no’ or ‘unclear’ = 0, generating an overall quality score for each study, which was used as a pragmatic summary indicator to facilitate team discussions around the quality of studies.

### Thematic synthesis

Thematic synthesis was chosen to allow for a rigorous and transparent synthesis of qualitative findings across studies, supporting the identification of recurring patterns and the generation of analytically themes relevant to the review aims. Thomas and Harden’s three stages of thematic synthesis were applied: coding the text line-by-line, developing descriptive themes and developing analytical themes [21]. Each paper was imported into NVivo 12. Line-by-line coding and a review of codes to find similarities to generate descriptive themes was conducted by JK. Descriptive themes were further refined by JK into analytical themes through an iterative process of comparing, merging and reinterpreting themes to identify overarching patterns and conceptual relationships. These themes were discussed with the wider review team (MA, GR, AW, TL), which included clinical and non-clinical academics with experience in self-management, primary care, healthy ageing and inequalities.

### Patient and public involvement members

Three patient and public involvement (PPI) contributors were involved in this review. All contributors were informal carers of people with dementia in deprived urban areas, providing them with relevant lived experience that aligns with the review’s focus. PPI members were involved in refining themes. JK met with the group to present initial themes and associated codes, which generated discussion around the relevance and resonance of each theme.

## Results

**Figure 1:**
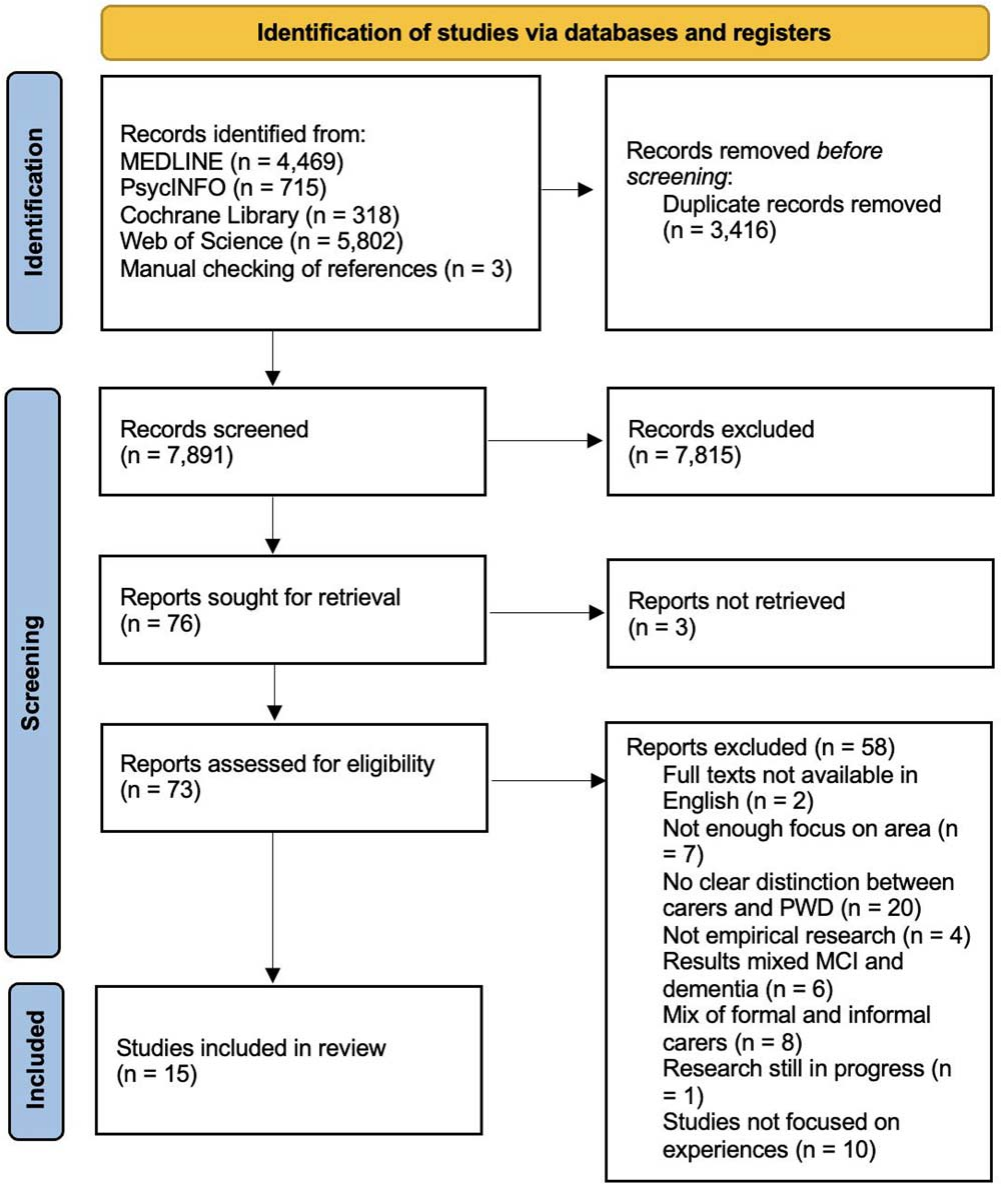
PRISMA flow diagram.

The initial search yielded 11 307 papers including three papers added through manual checking of references. A total of 7891 papers were screened after de-duplication. Fifteen studies were included in the review [22–36]. Figure  1 provides exclusion justifications at each stage.

### Screening agreement

Of the 3946 papers that were dual screened, 24 (0.6%) required further discussion. This reflected a very high level of agreement and consistency between reviewers and therefore, the authors did not believe that it was essential for the second reviewer to dual screen all papers.

### Study characteristics

Studies exploring people’s experiences of managing dementia in under-served geographical areas were based in the UK (n = 6), USA (n = 3), Canada (n = 2), Australia (n = 2), Hong Kong, (n = 1) and one study interviewed individuals in both England and The Netherlands.

Nine studies conducted interviews [22, 25, 27–30, 34–36], one study conducted focus groups [23] two studies carried out both focus groups and interviews [24, 31], two studies thematically analysed open-ended questionnaires [26, 33] and another conducted town hall meetings [32]. In total, 146 people with dementia, 573 informal carers and 127 key stakeholders, including physicians and advocates for people with dementia, participated in the included studies.

Two of the included studies were conducted within deprived areas, as defined by the authors of each study [25, 27]. Two other studies looked at both rural and urban areas where the urban area had not been defined as deprived, and therefore, was out of the scope of this research [22, 33]. All other studies (n = 11) were set only in rural areas. No studies were set in coastal areas. Participant characteristics from the included studies can be found in Table 1.

**Table 1:** Study characteristics.

Author, Year, Country	Title	Aim	Area (rural, coastal, deprived)	Research Design	Participants	Key findings
Bauer, Fetherstonhaugh, Blackberry, Farmer & Wilding (2019), Australia	*Identifying support needs to improve rural dementia services for people with dementia and their carers: A consultation study in Victoria, Australia*	To identify the support needs of people living with dementia and their carers, obtain stakeholder agreement on the top three priority support and service needs, and identify existing interventions, models of care or evidence-based services and supports available to respond to these needs.	Rural – defined as having a population density of 0.9 persons/km^2^ with half the population living outside the main towns (many with a population of <800 people).	Focus groups	3 PWD, 13 informal carers, 5 key informants. Genders and ethnicities of participants not reported.	Support and service needs of people with dementia and their carers are not always met in rural regions and there are significant unmet needs. Healthcare professionals are reluctant to make a formal diagnosis, and service providers require additional dementia-specific training.
Blackstock, Innes, Cox, Smith & Mason (2006), Scotland, UK	*Living with dementia in rural and remote Scotland: Diverse experiences of people with dementia and their carers*	To understand the positive and negative aspects of rural service provisions from service users’ and carers’ perspectives.	Rural – defined using the Scottish Executive Urban Rural Classification (2004).	Interviews and focus groups	15 PWD and 30 informal carers. 11/15 service users were female, and 22/30 carers were female. Ethnicities of participants not reported.	Participants identified both good and bad aspects to the support they received. They highlighted a lack of or poorly trained staff, poor communication, lack of information, lack of flexibility in service provision, and transport issues. 28 participants discussed community support compared to only 9 carers explicitly linking rural location to lack of services.
Chan, Chung, Ho-Wing Kwan & Woo (2022), Hong Kong	*Mitigating inequalities in community care needs of older adults with dementia: A qualitative case study of an integrated model of community care operated under the proportionate universalism principle*	To explore caregivers’ views on the experience of service use in the Hub and whether this service model is effective in mitigating social inequalities in community care needs of older adults with dementia and their own caregiving burden.	Deprived – defined by the level of co-payment required in the Community Care Service Voucher (CCSV) scheme which is based on household income. A higher subsidy indicates a lower household income.	Interviews	14 informal carers, 9 female and 5 male. Ethnicities of participants not reported.	The day care model of the Cadenza Hub addressed unmet needs of people with dementia and caregivers, including those of lower socioeconomic position. Improvements in self-care, functionality, social skills, and psychosocial well-being were observed. Carer stress relief was also noted.
Dal Bello-Haas, Cammer, Morgan, Stewart & Kosteniuk (2014), Canada	*Rural and remote dementia care challenges and needs: Perspectives of formal and informal care providers residing in Saskatchewan, Canada*	To describe formal and informal caregivers’ perceptions of challenges and needs associated with providing dementia care and support in rural and remote Saskatchewan.	Rural – defined Saskatchewan as rural as it covers an area of 651 036 km^2^ and has a population of ~1 million.	Thematic analysis of open-ended questionnaires	91 physicians, 151 informal carers. Genders and ethnicities of participants not reported.	Lack of services results in family caregivers acting as long-term care, increasing burden. Healthcare workers cited challenges providing services, especially in high Aboriginal population areas, due to lack of funding for travel and translation. 83% of physicians and 91% of non-physician healthcare workers wanted more dementia education. Caregivers reported long wait times and minimisation of memory problems by physicians.
Giebel, Robertson, Beaulen, Zwakhalen, Allen & Verbeek (2021), England, UK & The Netherlands	*‘Nobody Seems to Know Where to Even Turn To’: Barriers in Accessing and Utilising Dementia Care Services in England and The Netherlands*	To explore potential inequalities in access to formal dementia care services between England and the Netherlands, specifically from disadvantaged areas.	Deprived—no definition provided.	Interviews	13 PWD and 13 informal carers. Genders and ethnicities of interviewees not provided.	Most services accessed were self-funded. People with dementia and carers in the Netherlands were better supported by networks and case managers, which was less common in the UK.
Gulline, Carmody, Yates, Bevins, Brodtmann, Loi, Yin Lim, Macklin, Glennen, Woodward, Ayton & Ayton (2025), Australia	*Equity of access in rural and metropolitan dementia diagnosis, management and care experiences: An exploratory qualitative study*	To examine equity of access to dementia diagnosis, management and care services amongst people who experienced the process of dementia diagnosis as a patient or significant other across rural and metropolitan Australia.	Rural and urban – rurality defined by the Australian Statistical Geography Standard Remoteness Areas (ASGS-RA) and the Modified Monash Model (MMM).	Interviews	11 PWD, 26 informal carers. 12/14 rural participants were female. 16/23 metropolitan participants were female. Ethnicities of participants not reported.	Both urban and rural participants reported challenges with appointment wait times and navigating healthcare systems. Rural living added issues such as limited-service choice and lack of dementia-specific knowledge amongst healthcare professionals.
Herron & Rosenberg (2016), Canada	*‘Not there yet’: Examining community support from the perspective of people with dementia and their partners in care*	To explore how people with dementia connect with and contribute to communities, negotiate space outside the home, and identify desired support services.	Rural—defined by authors as a social construct grounded in material and geographic realties, economic factors and perceived rural identity.	Interviews	46 PWD, 43 informal carers, 26 males with dementia and 20 females with dementia. Genders of carers was not reported. Ethnicities of PWD and carers was not reported.	People with dementia identified needing companionship more than specific services. Many felt isolated and expressed aversion to group support.
Hicks, Innes & Nyman (2021), England, UK	*Experiences of rural life amongst community-dwelling older men with dementia and their implications for social inclusion*	To examine experiential accounts of older men with dementia as they negotiate social inclusion within rural communities.	Rural – locations chosen using the 2011 Rural–Urban Classification (DEFRA, 2015).	Interviews	17 males with dementia. All men were White British.	Men largely reported being content living with dementia in rural communities, emphasising positive community experiences and contrasting with the typical ‘tragedy discourse’. They appeared financially secure and able to live well with dementia.
Innes, Blackstock, Mason, Smith & Cox (2005), Scotland, UK	*Dementia care provisions in rural Scotland: Service users’ and carers’ experiences*	To develop a qualitative understanding of service use from the perspective of people with dementia and carers in rural Scotland.	Rural - defined using the Scottish Executive Urban Rural Classification (2003) where rurality is defined as settlements with ˂3000 people and drive times at least 30-min drive from a town with ˃10 000 people).	Interviews and focus groups	15 PWD and 16 informal carers interviewed. 14 different informal carers participated in focus groups. 11/15 PWD were female and 22/30 carers were female. Ethnicities of participants were not reported.	44 of 45 participants identified positive aspects of service use. 35 identified gaps in transport, respite care, carer support, home care and day care services.
Innes, Szymczynska & Stark (2014), Scotland, UK	*Dementia diagnosis and post-diagnostic support in Scottish rural communities: Experiences of people with dementia and their families*	To explore difficulties and satisfactions with diagnostic processes and post-diagnostic support for people with dementia and families in rural Scotland.	Rural – not explicitly defined but participants’ geographical location was categorised in the same way as previous work in Scotland by Innes *et al*. [31] and Blackstock *et al*. [24] using the Scottish Executive Urban Rural Classification.	Interviews	6 PWD, 12 informal carers 3/6 PWD were female. 11/12 carers were female. Ethnicities of participants were not reported.	Many carers initially denied issues, attributing them to ageing. Participants reported long waits for diagnosis and lack of post-diagnostic information or support.
Longstreth, McKibbin, Steinman, Slosser Worth & Carrico (2022), USA	*Exploring Information and Referral Needs of Individuals with Dementia and Informal Caregivers in Rural and Remote Areas*	To identify information and service needs, and preferences for delivery of information and referral resources amongst rural and remote dementia care recipients and caregivers.	Rural - defined through rural–urban continuum codes.	Town hall meetings	175 informal carers. 126 females. Ethnicities of participants not reported.	Findings highlight the importance of a single point of access for information/referral, local presence and ongoing assessment and information provision across the dementia care journey.
Roberts, Windle, MacLeod, Sullivan, Camic, Stott, Brotherhood, Jackson & Crutch (2024), Wales, UK	*‘It’s a Postcode Lottery’: How Do People Affected by Dementia in Wales Experience Their Diagnosis and Post-Diagnostic Support, and How May These Be Improved?*	To explore whether the aims of the Dementia Action Plan (assessment, diagnosis, post-diagnostic support) are being realised across Wales.	Rural and urban – defined by respondent.	Thematic analysis of open-ended questionnaires	9 PWD, 45 informal carers. 32 females (3 PWD, 29 carers). All participants were White British (2 with missing data).	Support quality varies regionally, creating a ‘postcode lottery’. Service commissioning differences lead to unequal access and care quality, though some areas show good practice and development plans.
Russell, Miyawaki, Reckrey & Bouldin (2024), USA	*Unmet Needs and Factors Impacting Home and Community-Based Service Use Amongst Rural Appalachian Caregivers of People with Alzheimer’s and Dementia*	To identify unmet needs and barriers to home and community-based healthcare services amongst rural Appalachian caregivers.	Rural – No definition provided.	Interviews	1 PWD and 21 informal carers. 80% female and 100% non-Hispanic white.	Carers had unmet needs around information, service navigation and caregiving support. Service use was shaped by illness needs, cultural beliefs and limited place-based resources.
Wallcook, Malinowsky, Charlesworth, Ryd & Nygard (2024), England, UK	*Everyday technology’s interplay in the lives of people with dementia: A multiple case study in the rural North of England*	To highlight the interplay between ET and everyday life situations amongst people living with dementia in a rural part of the North of England.	Rural – defined through population density.	Interviews	10 PWD. 4 female, ethnicities of participants not reported.	Technology is helpful but confusing to use. Some of the older people did not encourage or welcome this change whilst others embraced it and saw the technology as an increased social interaction and closeness/accessibility to family. One person in particular welcomed online shopping as he had to travel far to his local shop. However, another individual had little interest in upgrading his technology from a landline phone to a computer, and felt he wanted to cause as little trouble for his family in setting it up/teaching him as possible.
Waymouth, Siconolfi, Friedman, Saliba, Ahluwalia & Shih (2023), USA	*Barriers and Facilitators to Home and Community-based Services Access for Persons with Dementia and Their Caregivers*	To identify barriers and facilitators to home/community-based service (HCBS) access and how these contribute to disparities for persons with dementia living in rural areas and exacerbate disparities for minoritized populations.	Rural – no definition provided.	Interviews	31 stakeholders including HCBS providers and advocates for people with dementia – no participant characteristics reported.	Barriers included infrastructure, interpersonal, and individual-level challenges. Facilitators included dementia-specific practices, caregiver support and culturally competent education and services.

### Quality assessment

All methodologies from the included studies aligned with the analysis of data and interpretation of result and the conclusions drawn from each study flowed from the analysis of the data. However, no study directly addressed the influence of the researcher on the research. The full critical appraisal can be found in Table 2.

**Table 2:** JBI critical appraisal checklist for qualitative research.

Authors	Q1: Congruity between philosophical perspective and research methodology?	Q2: Congruity between methodology and the research question or objectives?	Q3: Congruity between methodology and the methods used to collect data?	Q4: Congruity between methodology and the representation and analysis of data?	Q5: Congruity between methodology and interpretation of results?	Q6: Statement locating the researcher culturally or theoretically?	Q7: influence of the researcher on the research, and vice-versa, addressed?	Q8: Are participants, and their voices, adequately represented?	Q9: Is the research ethical, or, for recent studies, is there evidence of ethical approval by an appropriate body?	Q10: Do the conclusions drawn in the report flow from the analysis, or interpretation of the data?	Overall Score
Bauer *et al*. [23]	Yes	Yes	Yes	Yes	Yes	No	No	Yes	Yes	Yes	8/10
Blackstock *et al*. [24]	Yes	Yes	Yes	Yes	Yes	No	No	Yes	Yes	Yes	8/10
Chan *et al*. [25]	Unclear	Yes	Yes	Yes	Yes	No	No	Yes	Yes	Yes	7/10
Dal Bello-Haas *et al*. [26]	Yes	Yes	Yes	Yes	Yes	No	No	Yes	Yes	Yes	8/10
Giebel *et al*. [27]	Yes	Yes	Yes	Yes	Yes	No	No	Yes	Yes	Yes	8/10
Gulline *et al*. [22]	Yes	Yes	Yes	Yes	Yes	No	No	Yes	Yes	Yes	8/10
Herron & Rosenberg [28]	Yes	Yes	Yes	Yes	Yes	No	No	Yes	Yes	Yes	8/10
Hicks *et al*. [29]	Yes	Yes	Yes	Yes	Yes	No	No	Yes	Yes	Yes	8/10
Innes *et al*. [31]	Yes	Yes	Yes	Yes	Yes	No	No	Yes	Yes	Yes	8/10
Innes *et al*. [30]	Yes	Yes	Yes	Yes	Yes	No	No	Yes	Yes	Yes	8/10
Longstreth *et al*. [32]	Yes	Yes	Yes	Yes	Yes	No	No	Yes	Yes	Yes	8/10
Roberts *et al*. [33]	Yes	Yes	Yes	Yes	Yes	No	No	Yes	Yes	Yes	8/10
Russell *et al*. [34]	Yes	Yes	Yes	Yes	Yes	No	No	Yes	Yes	Yes	8/10
Wallcook *et al*. [35]	Yes	Yes	Yes	Yes	Yes	No	No	Yes	Yes	Yes	8/10
Waymouth *et al*. [36]	Yes	Yes	Yes	Yes	Yes	No	No	Yes	Yes	Yes	8/10

### Data synthesis

Although 13 of the 15 studies were set in rural areas, two studies [25, 27] conducted their research within deprived areas, and therefore, only theme one is rural-specific. Other themes include codes from studies in deprived areas and are therefore relevant to those living in both rural and deprived areas. No studies were conducted in coastal areas, so those experiences are not included in these results. Eleven of the 15 included studies gathered data directly from people with dementia.

## Theme one: navigating stigma, privacy and disclosure in rural areas

Five studies described the stigma of dementia in rural areas [22, 24, 28, 32, 36]. Participants described the close-knit nature of rural communities as intensifying concerns of stigma. As people in rural areas often knew each other well, people with dementia felt more exposed to judgement and gossip, which heightened their reluctance to disclose symptoms or seek help.

This fear of judgement led several people with dementia to not disclose their diagnosis for fear of community rejection [28] with one participant stating she did not want her community thinking she was *‘nuts’* [28, p.84, PWD]. Another participant described her community’s reaction to her dementia diagnosis:

“Oh my gosh, Alzheimer’s stay away, it’s contagious” [28, p.84, PWD, rural area, Canada].

Other participants delayed seeking dementia-related medical care because of their personal relationships with their healthcare professionals [22].

“I was waiting for [someone] to come from out of the area and I waited for six months and then he didn’t come in the end” [20, p.8, PWD, rural area, Australia].

Dialogue around stigma often appeared alongside discussions of privacy and self-reliance when managing dementia in rural areas. For example, one informal carer discussed their experience of having a formal carer who lived in their village:

“The carer I had lives across the road and I knew her, I knew her family. And that can be a disadvantage, some people don’t like people that close knowing all about your family” [24 p.170, carer, rural area, Scotland]*.*

In one study, several participants stated that they were ‘not there yet’ when discussing the use of formal support and services [28, p.84, rural area, Canada], instead opting to manage dementia privately.

## Theme two: navigating fragmented healthcare systems and services

In rural contexts, people with dementia and their informal carers identified confusing referral pathways and a lack of local healthcare professionals with dementia knowledge as compounding unclear healthcare systems [22, 33]. These findings were substantiated by Dal Bello-Haasm *et al*. who reported that only 10% of rural physicians felt ‘extremely comfortable’ diagnosing and managing patients with dementia, whilst 83% felt they would benefit from additional dementia training [26]. Informal carers also related difficulties accessing, recruiting and retaining formal support to rurality [24]. As one informal carer stated:

“This geriatrician was only coming once a month and he was heavily booked out so that was going to take about three or four months before you could get an appointment” [20, p.4, carer, rural area, Australia].

Together, these barriers contributed to overly complicated journeys to diagnosis, including delayed or misdiagnosis, inconsistent follow-up and less perceived support [31, 33].

Participants across rural and deprived areas reported a reliance on their informal networks, or themselves, to try and source information, rather than relying on healthcare professionals [26, 27]. One informal carer stated:

“I’ve had to get my own information and have asked the doctor to act on it” [26, p.7, carer, rural area, Canada].

Despite being provided considerable information following a diagnosis, many still reported uncertainty about what services were available to them [22, 27, 32, 36]. Several people with dementia and informal carers found the volume of information and leaflets provided as overwhelming and confusing, especially directly following the diagnosis [27, 33].

## Theme three: lack of appropriate and accessible services

Participants in seven studies [27–31, 34, 36] discussed the unsuitability of services, expressing these did not reflect the interests and culture of their communities. As one participant stated, ‘support groups are not a normal rural solution’ [28, p. 85, rural area, Canada]. Those who did consider using services found they needed tailoring. One person with dementia felt that available day programme activities such as writing poetry were ‘emasculating’ [28]. Another person with dementia stated that available services ‘[didn’t] do much for me’ [29, p.453, PWD, rural area, England] as the activities within these services were too tedious. One carer stated:

“The only thing he has been offered is to go to these coffee mornings. He doesn’t want it and I’m not forcing him to go” [27, p. 9 carer, deprived area, England].

Due to a lack of appropriateness, several participants across rural and deprived areas opted not to use formal support services available in their area [27, 29, 30]. Others in rural areas reported barriers to accessing the support including a lack of government or personal funds to travel, public transport only available on certain days and reduced taxi services [26]. Where dementia-specific training was provided for drivers, one carer stated, ‘it’s a big relief to have somebody sitting with him. I can relax at work rather than thinking “what is going on?”’ [31, p.362, carer, rural area, Scotland]. However, such training was only discussed in one study.

## Theme four: positive experiences of living well with dementia

Five studies highlighted positive experiences of managing their dementia in rural and deprived areas [22, 27, 29, 30, 33]. Most of the positive experiences of managing dementia within the included papers were recalled by informal carers. These experiences encompassed multiple aspects of care including relationships with service providers and community support. Several carers described positive, efficient and supportive deliveries of dementia diagnoses [33] as well as how the diagnosis enabled access to higher quality and more diverse support [22, 27] One carer reflected:

*“*Ever since she officially received the diagnosis, many doors have opened. So that is just … just really nice” [27, p.10, carer, deprived area, The Netherlands].

Interactions with healthcare professionals were also a key factor in having a positive experience of managing dementia. Rural participants discussed positive experiences of efficient, friendly and knowledgeable healthcare professionals [22, 31]. One rural informal carer discussed how they maintained a relationship with local service providers beyond the care sessions, stating:

“Here it is different, you are always in contact….there is a friendship as well as caring” [24, p.168, carer, rural area, Scotland].

Several participants with dementia also felt their good relationships accounted for better and more personalised care [24].

Beyond formal support, informal carers highlighted how rural communities helped with watching out for people with dementia and with transport, running errands and helping with domestic duties [24].

### P‌PI contribution to theme development

The PPI members agreed with the interpretation of the themes and that the findings included within this review were the key findings. PPI members also discussed how the themes related to their own experiences. In theme two, the PPI group discussed the importance of highlighting how fragmented and confusing systems and services can place responsibility on carers and people with dementia themselves to source information, which was subsequently emphasised within this theme.

## Discussion

This review synthesised qualitative evidence on the experiences of people living with dementia in under-served areas in the UK, Australia, Hong Kong, USA and Canada. The 15 included studies revealed shared barriers to managing dementia across deprived and rural areas, primarily related to limited suitability, availability and accessibility of services and support. Despite variations in how rural life is characterised across countries, the studies reported broadly similar challenges in accessing and navigating dementia care. A key finding of this review was the central role of stigma in shaping experiences of managing dementia, a theme under-examined in similar reviews [3, 37]. Current literature highlights several strategies on how stigma could be reduced in rural areas. These include providing opportunities to learn and ask questions, challenging assumptions and delivering community-based educational workshops [38]. These recommendations align with our findings, which show that many rural residents lack dementia knowledge, with some treating the condition as though it were ‘contagious’ [28, p. 84, rural area, Canada]. However, although wider literature also advocates for rural support groups, this review suggests that such groups are not always appropriate in rural settings due to limited resident interest. This does not negate the potential value of support groups in rural communities, but indicates that interest and engagement may increase if other methods of stigma reduction were first used to begin reducing perceived and actual stigma in these communities.

In rural areas, this review found stigma of dementia as limiting help-seeking and social participation, which corresponds with evidence from other rural health contexts. In rural communities, the stigma of men having sex with other men was found to heighten internalised homophobia and lower self-esteem [39]. In another study, rural parents delayed or refrained from accessing mental health services for their children for fear of being judged as ‘bad parents’ [40]. Interestingly, within this review, rural community ties were beneficial to carers’ sense of security [24], helping to buffer carer burden. Existing literature reports increased carer burden and loneliness within deprived areas [41], though it remains unclear if community ties offer a similar level of protection within deprived settings. These findings highlight the complexity of place-based experiences, where protective factors for carers do not always translate into positive experiences for those living with dementia.

Difficulties in navigating healthcare systems resulted in some people with dementia missing out on services [22] and, in some cases, contributed to slower or misdiagnoses [22]. These findings align with a recent systematic review which stated that health and care services need to be more suitable for rural-dwelling people with dementia [3]. Several challenges identified around healthcare systems and services reflect recognised issues in managing dementia more widely [42]. Our findings show that in under-served settings, issues with finding and retaining formal care, infrequent visits from overstretched specialists, and lower health literacy can intensify challenges in dementia care. Therefore, services need to go beyond generic approaches to instead consider the compounded impact of place-based experiences such as socioeconomic deprivation and stigma.

Suitability of services impacted experiences of social isolation and unmet support needs [27, 28]. Across both rural and deprived settings, sparse service provisions meant that people with dementia were frequently offered only a single type of support, foregrounding the importance of the suitability of services within specific community contexts. In several studies, how well aligned available services were with local community values, cultural expectations and individual preferences emerged as a central factor shaping service uptake. Where services did not resonate with locals, such as group-based interventions being offered in communities that valued privacy and self-reliance, people with dementia were less likely to engage. Although many existing community services were viewed as unsuitable or unappealing, our findings do not suggest that place-based approaches are ineffective. Rather, they highlight that they currently fail to reflect the preferences and constraints of under-served communities. The lack of interest expressed in the local support appeared to stem from issues of relevance, accessibility and stigma, rather than a rejection of local support completely. These findings are consistent with existing research on the importance of tailoring primary health care models [43] and locally informed services [44] to meet the needs of rural populations. Our findings highlight the importance of tailored services in socioeconomically deprived populations too.

## Strengths and limitations of the review

This review offers a comprehensive synthesis of qualitative evidence on the experiences of people living with dementia in underserved areas across eight countries. A key strength of this review is its focus on multiple place-based disparities which are under-examined in existing dementia reviews. The use of a transparent approach to searching, screening and analysis enhances the credibility of findings.

We had 50% of studies at the title and abstract stage double-screened, although predefined criteria and discussion of uncertainties within the wider review team helped to mitigate potential bias. Additionally, although our search strategy was thorough, we were unable to conduct grey literature searches, which may have limited the identification of community-based reports or local evaluations relevant to underserved areas. Furthermore, PPI input for this study was provided only by informal carers of people with dementia, which could have refined themes in a way that reflects carer experiences more than those of people with dementia. Finally, most of the studies were conducted in high-income, English-speaking countries, which restricts the transferability of the findings to other global settings.

## Implications for future research and clinical practice

Future research should seek to understand how people living in coastal areas manage their dementia, particularly given the growing recognition of health inequalities in these communities [13]. This research should also explore whether the challenges and unmet needs of people with dementia differ between mainland coastal populations and those living on islands, as these contexts may present distinct barriers to support and service access. Although our review aimed to examine experiences across a range of under-served settings, no studies focused on coastal areas and relatively few explored the experiences of people living with dementia in deprived communities. This evidence gap highlights an important research priority: the need for studies that examine how coastal or deprived areas shape dementia experiences, service access and stigma.

Research should also explore how different geographic and socioeconomic factors intersect to impact individuals’ experiences of managing dementia. This synthesis suggests that people living in rural and some deprived areas face additional challenges, but the included studies rarely reported intersecting characteristics, such as race, ethnicity, sexuality or religion, limiting our ability to empirically examine them. Future research would benefit from exploring if, or how, these intersections further influence how people manage dementia in under-served areas.

This review has highlighted the need for more widespread person-centred care. Future services should be co-produced with people who will be utilising them and should differ by area and needs. Health systems should adopt place-based dementia strategies that recognise local barriers (e.g. transport, digital exclusion) and health and social care providers should develop flexible service delivery models (e.g. mobile clinics, telehealth, outreach visits) to mitigate geographic barriers.

## Conclusions

This review highlighted that people living with dementia in under-served rural and deprived areas face persistent barriers to living well due to limited service suitability, availability and accessibility, as well as stigma on disclosure and help seeking. Whilst informal carers often viewed closeknit rural communities as protective, people with dementia frequently experienced these same dynamics as increasing potential judgement. Challenges navigating healthcare systems, exacerbated by limited professional signposting, digital exclusion and travel constraints, contributed to delayed diagnoses and unmet support needs. These findings highlight the need for place sensitive services that reflect local values and tackle place-specific barriers including addressing stigma and simplifying care pathways. Evidence gaps remain for coastal areas, underscoring the importance of research examining how place, deprivation and social identity intersect to shape dementia care experiences.

## Supplementary Material

aa-26-0609-File002_afag190

## References

[1] World Health Organization . Dementia. Geneva: World Health Organisation [Internet], 2025, [cited 2025 Oct 30]. Available from: https://www.who.int/news-room/fact-sheets/detail/dementia.

[2] An Evidence Summary of Health Inequalities in Older Populations in Coastal and Rural Areas Full Report [Internet], 2019, [cited 2026 Apr 21]. Available from: https://assets.publishing.service.gov.uk/media/5d517ce3ed915d7646dea423/Health_Inequalities_in_Ageing_in_Rural_and_Coastal_Areas-Full_report.pdf.

[3] Giebel C, Readman MR, Godfrey A, Gray A, Carton J, Polden M. Geographical inequalities in dementia diagnosis and care: a systematic review. International Psychogeriatrics. 2025;37:100051. 10.1016/j.inpsyc.2025.100051 PMC12149024 39986949

[4] Older People JB, Branch D . National Dementia Strategy. Living well with dementia: a National Dementia Strategy. Title Living well with dementia: a National Dementia Strategy.

[5] House of Lords Library . *Health care in rural areas**.* London: House of Lords Library [Internet], 2023,[cited 2026 Jan 8]. Available from: https://lordslibrary.parliament.uk/health-care-in-rural-areas/.

[6] Rahman M, White EM, Mills C et al. Rural-urban differences in diagnostic incidence and prevalence of Alzheimer’s disease and related dementias. Alzheimer’s Dementia 2021; 17:1213–30. 10.1002/alz.12285 PubMed PMID: 33663019. PMC8277695 33663019

[7] Watson J, Green MA, Giebel C et al. Social and spatial inequalities in healthcare use among people living with dementia in England (2002-2016). Aging Ment Health 2023;27:1476–87. 10.1080/13607863.2022.2107176. 35959941 PMC9612936

[8] Shah S, Syed R, Khan AA et al. Urban–rural disparities in dementia-related mortality among older adults in the U.S. from 1999 to 2020. Archives Gerontol Geriatrics Plus 2025;2:100214. 10.1016/J.AGGP.2025.100214.

[9] NHS England . Deprivation [Internet], [cited 2026 Jan 19]. Available from: https://www.england.nhs.uk/about/equality/equality-hub/national-healthcare-inequalities-improvement-programme/what-are-healthcare-inequalities/deprivation/.

[10] Low A, Tsvetanov KA, Ntailianis G et al. Neighborhood deprivation and midlife cognition: evidence of a modifiable vascular pathway involving health behaviors and cerebral small vessel disease. Alzheimers Dement 2025;21:e70756. 10.1002/alz.70756. 41190623 PMC12587304

[11] Petersen JD, Wehberg S, Packness A et al. Association of socioeconomic status with dementia diagnosis among older adults in Denmark. JAMA Netw Open 2021;4:E2110432. 10.1001/jamanetworkopen.2021.10432 PubMed PMID: 34003271. 34003271 PMC8132141

[12] Alzheimer’s Research UK . Health-Inequalties-Full-Report, 2023.

[13] Chief Medical Officer’s Annual Report 2021 . Health in Coastal Communities – Summary and Recommendations.

[14] Coastal Towns in England and Wales - Office for National Statistics [Internet], [cited 2025 Nov 6]. Available from: https://www.ons.gov.uk/businessindustryandtrade/tourismindustry/articles/coastaltownsinenglandandwales/2020-10-06.

[15] Age UK PHE . Exploring the Factors Underlying Health Inequalities for Older Men, Older People from Ethnic Minorities, and Older LGBTQ+ People Ageing in Coastal and Rural Communities, 2019.

[16] Ward R, Clark A, Campbell S et al. The lived neighborhood: understanding how people with dementia engage with their local environment. Int Psychogeriatr 2017;30:867–80. 10.1017/S1041610217000631. 28462764 PMC6088530

[17] Garrett MH, Azar D, Goeman D et al. Health and social care needs of people living with dementia: a qualitative study of dementia support in the Victorian region of Gippsland, Australia. Rural Remote Health 2024;24:8244. 10.22605/RRH8244 PubMed PMID: 38233335. 38233335

[18] Hicks B, Wheatley K, Porter E et al. A mapping review of studies exploring the barriers and facilitators to a dementia diagnosis through an intersectionality lens. BJPsych Open 2025;11:e76. 10.1192/BJO.2025.17. 40214113 PMC12052574

[19] Page MJ, McKenzie JE, Bossuyt PM et al. The PRISMA 2020 statement: an updated guideline for reporting systematic reviews. BMJ. 2021;372:71. 10.1136/bmj.n71. PMC8005924 33782057

[20] JBI Critical Appraisal Tools | JBI [Internet], [cited 2025 Dec 9]. Available from: https://jbi.global/critical-appraisal-tools.

[21] Thomas J, Harden A. Methods for the thematic synthesis of qualitative research in systematic reviews. BMC Med Res Methodol 2008;8:8. 10.1186/1471-2288-8-45 PubMed PMID: 18616818. 18616818 PMC2478656

[22] Gulline H, Carmody S, Yates M et al. Equity of access in rural and metropolitan dementia diagnosis, management, and care experiences: an exploratory qualitative study. Int J Equity Health 2025;24:74. 10.1186/s12939-025-02434-1 PubMed PMID: 40091013. PMC11912628 40091013

[23] Bauer M, Fetherstonhaugh D, Blackberry I et al. Identifying support needs to improve rural dementia services for people with dementia and their carers: a consultation study in Victoria. Australia Australian J Rural Health 2019;27:22–7. 10.1111/ajr.12444. 30719789

[24] Blackstock KL, Innes A, Cox S et al. Living with dementia in rural and remote Scotland: diverse experiences of people with dementia and their carers. J Rural Stud 2006;22:161–76. 10.1016/j.jrurstud.2005.08.007.

[25] Chan SM, Chung GKK, Kwan MHW et al. Mitigating inequalities in community care needs of older adults with dementia: a qualitative case study of an integrated model of community care operated under the proportionate universalism principle. BMC Primary Care 2022;23:244. 10.1186/s12875-022-01855-z. PMC9494773 36131245

[26] Dal Bello-Haasm V, Cammer A, Morgan D, Stewart N, Kosteniuk J, Bello-Haas VPM D. Rural and remote dementia care challenges and needs: perspectives of formal and informal care providers residing in Saskatchewan, Canada. *Rural and Remote* Health 2014;14:2747. 10.22605/RRH2747 25081857

[27] Giebel C, Verbeek H, Robertson S et al. “Nobody seems to know where to even turn to”: barriers in accessing and utilising dementia Care Services in England and the Netherlands. Int J Environ Res Public Health 2021;18:12233. 10.3390/ijerph182212233. PMC8622725 34831989

[28] Herron RV, Rosenberg MW. “Not there yet”: examining community support from the perspective of people with dementia and their partners in care. Soc Sci Med 2017;173:81–7. 10.1016/j.socscimed.2016.11.041. 27930919

[29] Hicks B, Innes A, Nyman SR. Experiences of rural life among community-dwelling older men with dementia and their implications for social inclusion. Dementia. 2021;20:444–63. 10.1177/1471301219887586. 31718267

[30] Innes A, Szymczynska P, Stark C. Dementia diagnosis and post-diagnostic support in Scottish rural communities: experiences of people with dementia and their families. Dementia. 2014;13:233–47. 10.1177/1471301212460608. 24599816

[31] Innes A, Blackstock K, Mason A et al. Dementia care provision in rural Scotland: service users’ and carers’ experiences. Health Soc Care Community 2005;13:354–65. 10.1111/j.1365-2524.2005.00569.x. 15969707

[32] Longstreth M, McKibbin C, Steinman B et al. Exploring information and referral needs of individuals with dementias and informal caregivers in rural and remote areas. Clin Gerontol 2022;45:808–20. 10.1080/07317115.2019.1710735 PubMed PMID: 31920164. 31920164

[33] Roberts JR, Windle G, MacLeod CA et al. “It’s a postcode lottery”: how do people affected by dementia in Wales experience their diagnosis and post-diagnostic support, and how may these Be improved? Int J Environ Res Public Health 2024;21:709. 10.3390/ijerph21060709. PMC11203760 38928955

[34] Russell D, Miyawaki CE, Reckrey JM et al. Unmet needs and factors impacting home- and community-based service use among rural Appalachian caregivers of people with Alzheimer’s and dementia. J Appl Gerontol 2025;44:628–37. 10.1177/07334648241280041. 39263814 PMC11896892

[35] Wallcook S, Malinowsky C, Charlesworth G et al. Everyday technology’s interplay in the lives of people with dementia: a multiple case study in the rural north of England. J Rural Stud 2024;106:103203. 10.1016/j.jrurstud.2024.103203.

[36] Waymouth M, Siconolfi D, Friedman EM et al. Barriers and facilitators to home- and community-based services access for persons with dementia and their caregivers. J Gerontol Ser B Psychol Sci Soc Sci 2023;78:1085–97. 10.1093/geronb/gbad039. 36896936 PMC10214645

[37] Arsenault-Lapierre G, Bui TX, Le Berre M et al. Rural and urban differences in quality of dementia care of persons with dementia and caregivers across all domains: a systematic review. BMC Health Serv Res 2023;23:102. 10.1186/s12913-023-09100-8 PubMed PMID: 36721162. 36721162 PMC9887943

[38] Bacsu J, Johnson S, O’Connell M et al. Interventions to reduce stigma of dementia: first insights from a rural community-based participatory study. Innov Aging 2020;4:879. 10.1093/GERONI/IGAA057.3247.

[39] Preston DB, D’Augelli AR, Kassab CD et al. The relationship of stigma to the sexual risk behavior of rural men who have sex with men. AIDS Educ Prev 2007;19:218–30. 10.1521/AEAP.2007.19.3.218. 17563276

[40] Boydell KM, Stasiulis E, Barwick M et al. Challenges of knowledge translation in rural communities: the case of rural Children’S mental. Health. 2008;27:49–63. 10.7870/CJCMH-2008-0004.

[41] Armstrong M, Jeri-Wahrhaftig A, Woodward A et al. Experiencing socioeconomic deprivation as a carer in the United Kingdom: a qualitative study. Health Expect 2025;28:e70502. 10.1111/HEX.70502. 41261758 PMC12630546

[42] Giebel C, Reilly S, Gabbay M et al. Dementia care navigation: a systematic review on different service types and their prevalence. Int J Geriatr Psychiatry 2023;38:e5977. 10.1002/GPS.5977. 37526320

[43] Morgan D, Kosteniuk J, Seitz D et al. A five-step approach for developing and implementing a rural primary health care model for dementia: a community–academic partnership. Prim Health Care Res Dev 2019;20:e29. 10.1017/S1463423618000968 PubMed PMID: 32799988. 32799988 PMC6536750

[44] LAK Wiese, A Gibson, MA Guest, AR Nelson, R Weaver, A Gupta et al. Global Rural Health Disparities in Alzheimer’s Disease and Related Dementias: State of the Science. Alzheimer’s and Dementia. 2023;19:4204–25. 10.1002/alz.13104. PMC10524180 37218539

